# Comparative transcriptional profiling of canine acanthomatous ameloblastoma and homology with human ameloblastoma

**DOI:** 10.1038/s41598-021-97430-0

**Published:** 2021-09-07

**Authors:** Santiago Peralta, Gerald E. Duhamel, William P. Katt, Kristiina Heikinheimo, Andrew D. Miller, Faraz Ahmed, Angela L. McCleary-Wheeler, Jennifer K. Grenier

**Affiliations:** 1 Department of Clinical Sciences, Clinical Programs Center, College of Veterinary Medicine, Cornell University, Box 31, Ithaca, NY 14853 USA; 2 Department of Biomedical Sciences, College of Veterinary Medicine, Cornell University, Ithaca, NY 14853 USA; 3 Department of Molecular Medicine, College of Veterinary Medicine, Cornell University, Ithaca, NY 14853 USA; 4 Department of Oral and Maxillofacial Surgery, Institute of Dentistry, University of Turku and Turku University Hospital, Turku, Finland; 5 Department of Veterinary Medicine and Surgery, College of Veterinary Medicine, University of Missouri, Columbia, MO 65211 USA

**Keywords:** Cancer models, Gene expression, Cancer genomics, Cancer models, Head and neck cancer, Oral cancer, Oncogenesis, Oncology

## Abstract

Ameloblastomas are odontogenic tumors that are rare in people but have a relatively high prevalence in dogs. Because canine acanthomatous ameloblastomas (CAA) have clinicopathologic and molecular features in common with human ameloblastomas (AM), spontaneous CAA can serve as a useful translational model of disease. However, the molecular basis of CAA and how it compares to AM are incompletely understood**.** In this study, we compared the global genomic expression profile of CAA with AM and evaluated its dental origin by using a bulk RNA-seq approach. For these studies, healthy gingiva and canine oral squamous cell carcinoma served as controls. We found that aberrant RAS signaling, and activation of the epithelial-to-mesenchymal transition cellular program are involved in the pathogenesis of CAA, and that CAA is enriched with genes known to be upregulated in AM including those expressed during the early stages of tooth development, suggesting a high level of molecular homology. These results support the model that domestic dogs with spontaneous CAA have potential for pre-clinical assessment of targeted therapeutic modalities against AM.

## Introduction

Ameloblastoma is an epithelial tumor of odontogenic origin that is rare in people, but relatively common in domestic dogs^1–4^. Despite its histologically benign features and a low metastatic potential, canine acanthomatous ameloblastoma (CAA) exhibits locally invasive behavior with a tendency to infiltrate adjacent structures including the jawbone, similar to human ameloblastoma (AM)^3–7^. Although e*n bloc* surgical excision is the current treatment of choice that allows long-term remission^4–7^, this is a technically complex and invasive procedure that often results in patient disfigurement and dysfunction^8–10^. By contrast, radiation therapy and marginal excision are less invasive options but are associated with a higher rate of persistence and recurrence^11–14^. Thus, novel therapeutic modalities, including small molecule tumor driver inhibitors, are attractive alternatives that may improve outcomes while minimizing side-effects. However, development of effective therapeutic approaches requires robust models of disease and identification of druggable targets associated with oncogenesis.

Recent discoveries by us and others reveal mutually exclusive RAS-RAF-MAPK pathway activating mutations suggesting conserved molecular mechanisms in AM and CAA^15,16^. The MAPK pathway, which regulates cell survival, is of major interest given that it is a widely used target for several different tumors expressing driving mutations^17,18^. Indeed, several small molecules have FDA approval for treatment of various types of cancers that harbor RAS-RAF-MAPK pathway activating mutations, e.g., colorectal cancer and melanoma. Current evidence also suggests that such approach may be effective for therapeutic management of AM^19–21^. However, in vivo models amenable to large scale clinical trials are lacking, thus making the relatively common spontaneous CAA an attractive pre-clinical model for these studies.

Since CAA has clinical, histomorphological, tumor biological, and mutational similarities to AM^16,22^, and compared to existing laboratory animal models, domestic dogs develop spontaneous tumors that recapitulate the environmental, genetic, immunological, metabolic, pharmacological, and clinical complexities of human oncogenesis^23–25^, CAA represents a suitable pre-clinical model to test novel therapies for AM. Moreover, the high incidence of CAA compared to AM provides a readily available patient population, while simultaneously offering client-owned dogs an opportunity to benefit from rational and potentially more effective, less detrimental interventions. However, aside from shared oncogenic mutations, the molecular basis of CAA and the degree of homology with AM are still incompletely known. Therefore, the aim of this study was to compare the global transcriptional profile of CAA to that of AM. For this, healthy gingiva (HGIN) and canine oral squamous cell carcinoma (COSCC) served as controls for non-tumoral tissue and a different oral neoplasm, respectively.

## Results

### Clinical samples

Study material consisted of cryopreserved fresh tissue samples and archived formalin-fixed paraffin-embedded (FFPE) tissues obtained from 37 client-owned dogs of variable age, breed, and sex, representing 20 CAA,12 COSCC, and 5 HGIN samples (Table 1, Supplemental Table S1). Of 37 samples available, 25 corresponding cryopreserved samples, representing 12 CAA, 8 COSCC and 5 HGIN samples, were profiled with RNA-seq. An additional 12 samples were used to complement RNA-seq data validation assays including reverse transcriptase quantitative PCR (qPCR) and immunohistochemistry (IHC) assays. The mutational profile of 28 of the 37 samples had previously been reported using a candidate gene approach^16^. Briefly, results showed that 15 of the 16 (93.8%) analyzed CAA samples harbored *HRAS* p.Q61R somatic mutations, 2 of the 8 (25%) analyzed COSCC analyzed harbored *BRAF* p.V600E somatic mutations, and 1 of the 8 (12.5%) analyzed COSCC samples harbored an *HRAS* p.Q61L somatic mutation.

**Table 1:** Case description and assays performed.

Case no.	Diagnosis	HRAS/BRAF status	Molecular assays		Immunohistochemistry		
			RNA-seq	qPCR	EMT markers*	pERK1/2**	ODAM**
1	CAA	*HRAS* p.Q61R	Yes	Yes	< 20%	ND	ND
2	CAA	*HRAS* p.Q61R	Yes	Yes	50%	++	ND
3	CAA	*HRAS* p.Q61R	No	Yes	ND	ND	ND
4	CAA	*HRAS* p.Q61R	Yes	Yes	90%	ND	+++
5	CAA	*HRAS* p.Q61R	Yes	Yes	ND	ND	ND
6	CAA	*HRAS* p.Q61R	Yes	Yes	50%	ND	ND
7	CAA	*HRAS* p.Q61R	Yes	Yes	ND	ND	ND
8	CAA	*HRAS* p.Q61R	Yes	Yes	ND	++	ND
9	CAA	*HRAS* p.Q61R	No	Yes	ND	ND	ND
10	CAA	*HRAS* p.Q61R	No	Yes	ND	ND	ND
11	CAA	*HRAS* p.Q61R	Yes	Yes	< 20%	ND	ND
12	CAA	*HRAS* p.Q61R	Yes	Yes	75%	++	ND
13	CAA	*HRAS* p.Q61R	Yes	Yes	ND	+++	ND
14	CAA	*HRAS* p.Q61R	Yes	Yes	50%	ND	+++
15	CAA	*HRAS* p.Q61R	No	Yes	ND	+++	ND
16	CAA	WT	Yes	Yes	90%	ND	ND
17	OSCC	WT	No	Yes	ND	ND	ND
18	OSCC	*BRAF* p.V600E	Yes	Yes	ND	++	ND
19	OSCC	WT	Yes	Yes	ND	ND	ND
20	OSCC	WT	Yes	Yes	ND	+	ND
21	OSCC	WT	Yes	Yes	ND	ND	ND
22	OSCC	*HRAS* p.Q61L	Yes	Yes	ND	+	ND
23	OSCC	WT	Yes	Yes	ND	+++	ND
24	OSCC	*BRAF* p.V600E	Yes	Yes	ND	ND	ND
25	HGIN	WT	Yes	Yes	ND	ND	ND
26	HGIN	WT	Yes	Yes	ND	ND	ND
27	HGIN	WT	Yes	Yes	ND	ND	ND
28	HGIN	WT	Yes	Yes	ND	ND	ND
29	CAA	ND	No	No	ND	++	ND
30	CAA	ND	No	No	ND	+++	ND
31	CAA	ND	No	No	ND	+++	ND
32	CAA	ND	No	No	ND	++	ND
33	OSCC	ND	No	No	ND	+++	ND
34	OSCC	ND	No	No	ND	+++	ND
35	OSCC	ND	No	No	ND	+++	ND
36	OSCC	WT	Yes	No	ND	ND	ND
37	HGIN	WT	Yes	No	ND	ND	ND

*Results are shown as a percentage of the area of each tumor exhibiting a staining pattern consistent with EMT, as described in the text.

**Symbols denote the intensity of the signal observed as follows: weak (+), intermediate (++), or strong (+++). Scores of IHC results were not compared due to limited number of cases available for staining.

Abbreviations: *CAA* canine acanthomatous ameloblastoma, *HGIN* healthy gingiva, *OSCC* canine oral squamous cell carcinoma, *EMT* epithelial-to-mesenchymal transition, *WT* wild type, *ND* not determined.

### Differential gene expression, cluster analysis and validation assays

**Figure 1:**
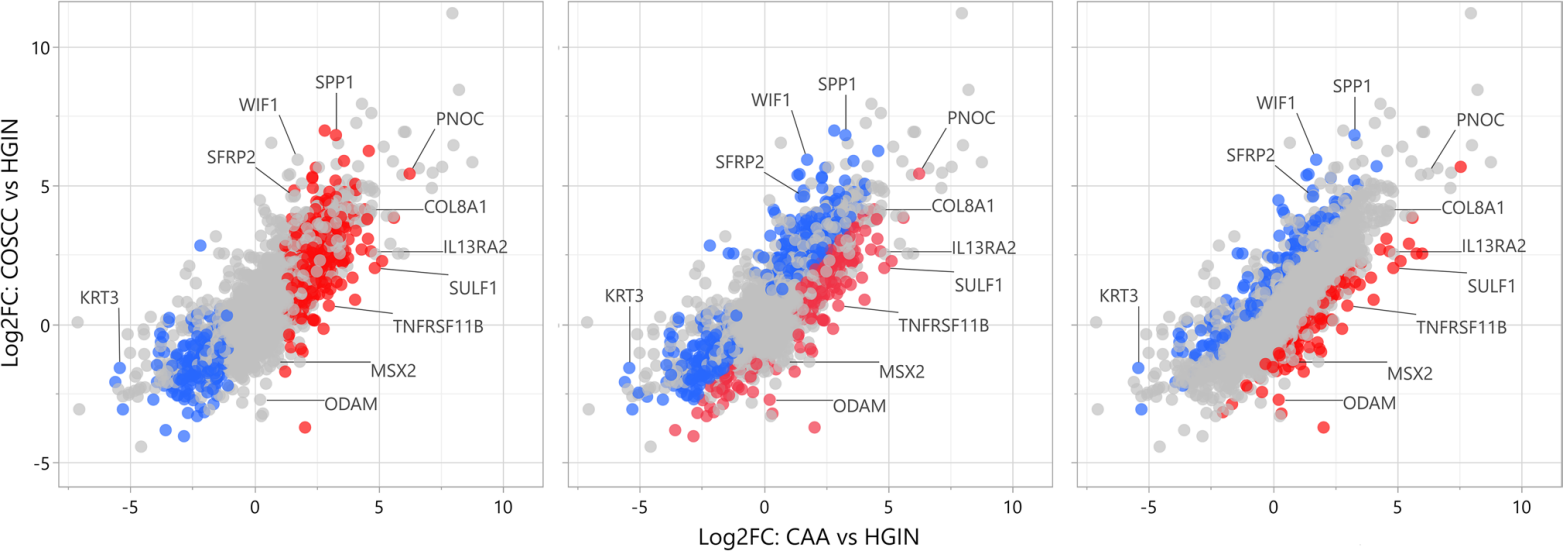
Scatterplots showing patterns of differential gene expression in CAA and COSCC according to log2-fold change (Log2FC) values relative to HGIN. Each point, regardless of color, corresponds to an individual gene; only expressed genes are depicted. The eleven labeled genes correspond to those that were used for qPCR validation assays. The three panels differ only by coloration of individual genes. Panel (**A**) shows 783 genes that were found to be significantly upregulated (red) and 412 that were downregulated (blue) in CAA compared to HGIN when applying stringent differential expression criteria. The colored (red and blue) points in panel B depict 1507 genes that were found to be differentially expressed in one or both tumors when compared to HGIN (i.e., CAA vs HGIN or COSCC vs HGIN), with red points corresponding to those that were upregulated in CAA versus COSCC, and blue points corresponding to those that were downregulated in CAA versus COSCC. Panel C shows 142 genes that were significantly upregulated (red) and 272 genes that were significantly downregulated (blue) in CAA versus COSCC regardless of how they compared to HGIN. Abbreviations: *HGIN* healthy gingiva, *OSCC* canine oral squamous cell carcinoma, *CAA* canine acanthomatous ameloblastoma.

**Figure 2:**
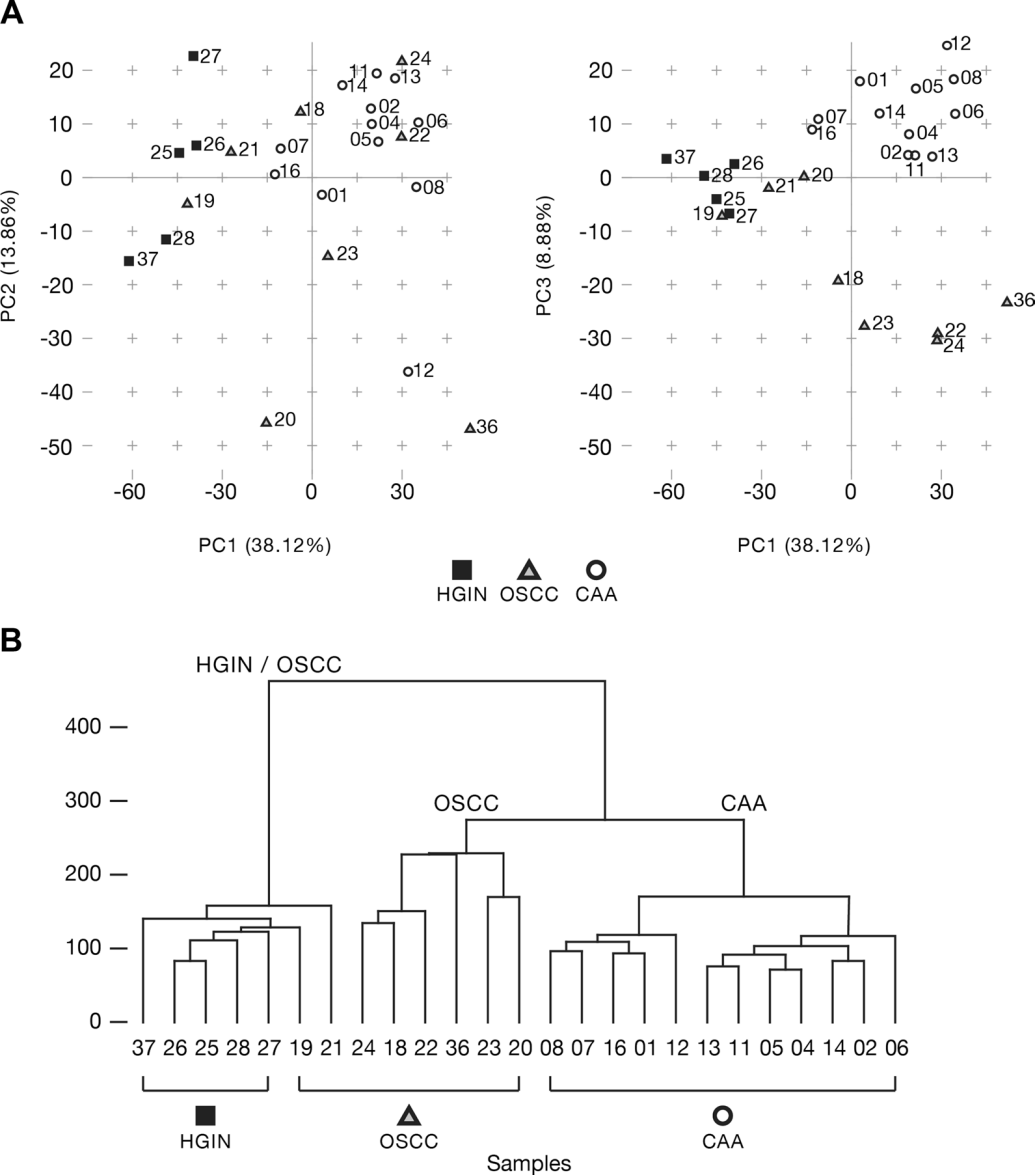
Unsupervised sample clustering. Principal component analysis (panel **A**) and hierarchical clustering (panel **B**) independently showed CAA clustering apart from COSCC and HGIN. Abbreviations: *HGIN* healthy gingiva, *OSCC* canine oral squamous cell carcinoma, *CAA* canine acanthomatous ameloblastoma.

**Figure 3:**
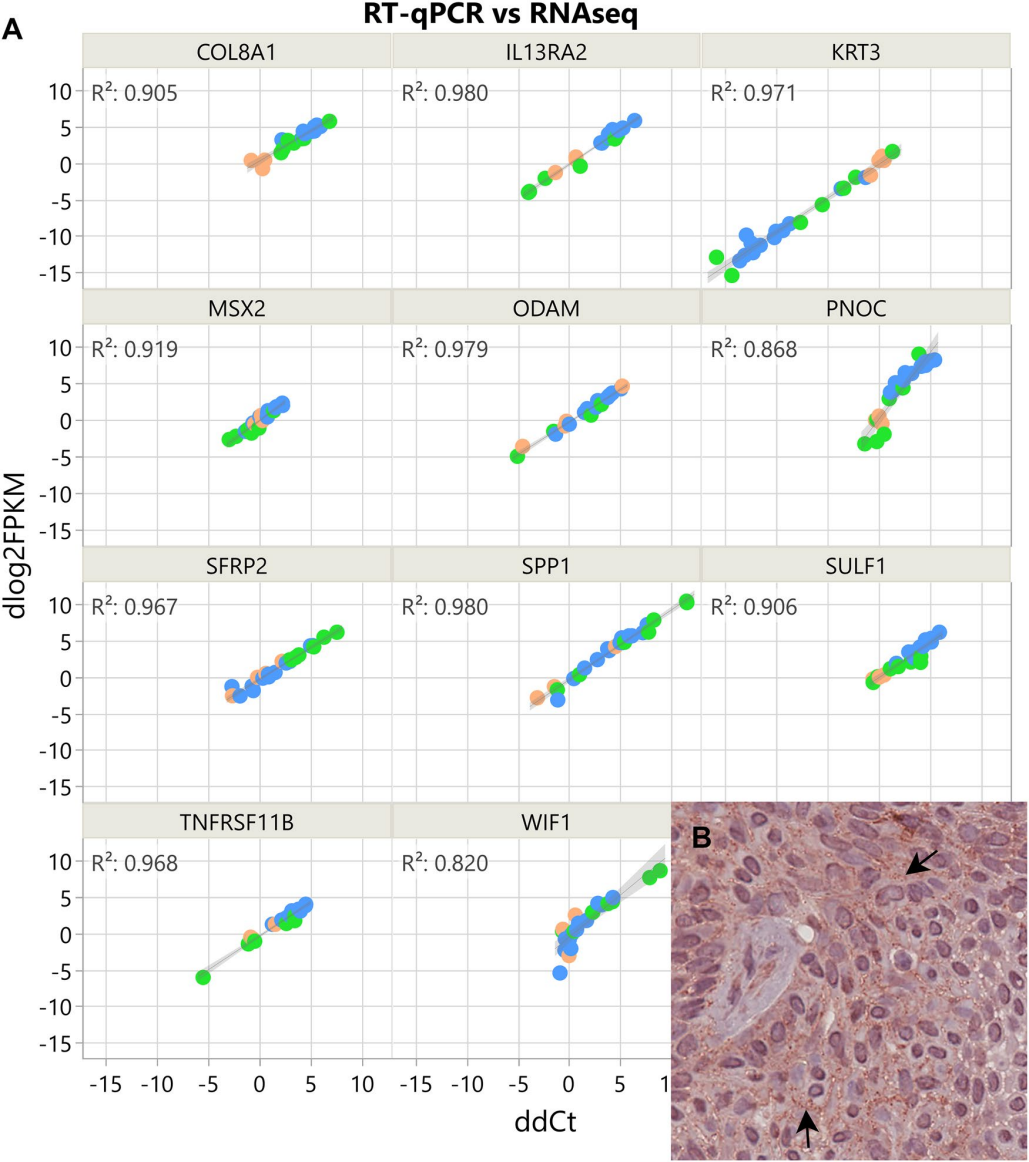
Results of qPCR and IHC validation assays. Panel (**A**) shows the results of qPCR assays (ddCt, X axis) of 11 genes, plotted against the results of RNA-seq (dlog2FPKM, Y axis), where dlog2FPKM is the difference between the log2(FPKM) value for each sample compared to the average log2(FPKM) for healthy gingiva control samples. The measurements with both gene expression quantification platforms were in excellent agreement for all phenotypes including CAA (blue), COSCC (green) and HGIN (orange); the points lying close to the line were best reproduced between the two experiments. Panel (**B**) corresponds to a × 40 photomicrograph showing cytoplasmic and nuclear ODAM immunoreactivity (black arrows) of proliferating neoplastic epithelium in one of the CAA cases analyzed.

Of the 24,580 annotated dog genes (CanFam3, Ensembl gene build), RNA-seq revealed 1,195 genes differentially expressed (q < 0.05) in CAA when compared to HGIN, 898 genes in OSCC compared to HGIN, and 414 genes in CAA when compared to COSCC (Fig. 1, Supplemental Tables S2–4). Principal component analysis and unsupervised hierarchical cluster analysis were performed using independent algorithms with all samples clustering according to tumor/tissue type (Fig. 2). To validate the RNA-seq profiles, 11 genes differentially expressed in at least one of the three pairwise comparisons (i.e., CAA vs HGIN, COSCC vs HGIN, and CAA vs COSCC) were selected for qPCR. Overall, excellent agreement between RNA-seq and qPCR results was found (Fig. 3A, Supplemental Figure S5), including extending the observation of altered gene expression for samples outside the RNA-seq cohort. For biological validation of *ODAM*, one of the differentially overexpressed genes in CAA, expression of the ODAM protein was assessed by IHC in two CAA samples. We found cytoplasmic and nuclear expression of ODAM in CAA tumor cells (Fig. 3B, Supplemental Figure S6). Moreover, ODAM expression was multifocal and without segregation to basal or suprabasilar neoplastic epithelial cells. Although protein expression was not confirmed with IHC in HGIN or COSCC samples, RNA-seq and qPCR results also showed *ODAM* to be relatively highly expressed in 1 of the 5 HGIN samples, and in 2 of the 8 COSCC samples (Supplemental Figure S5), demonstrating that its expression is not exclusive to CAA, as would be expected based on previous studies^26–28^.

### Functional enrichment analyses

**Figure 4:**
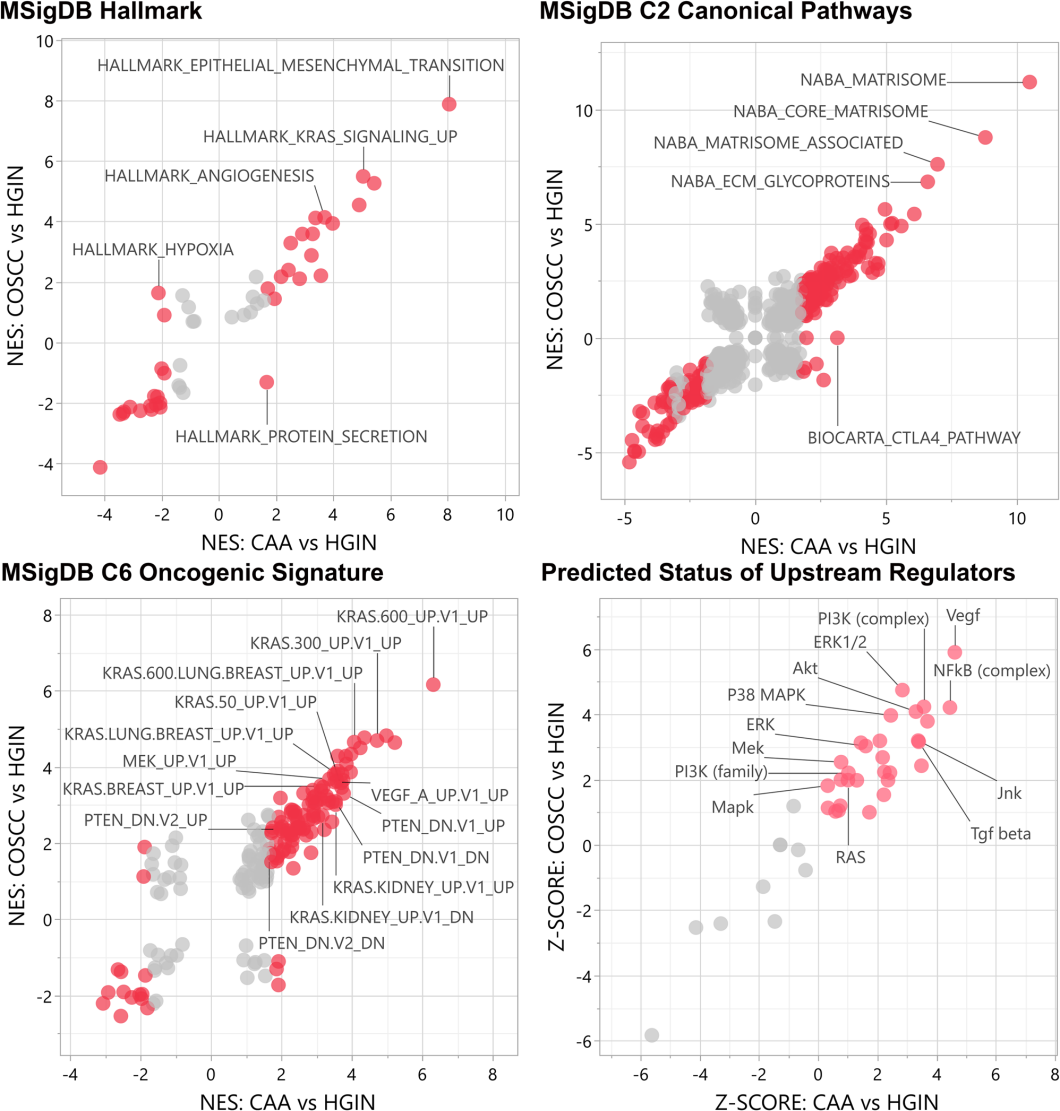
Functional enrichment analyses. The top two and the bottom left scatterplots depict normalized enrichment scores (NES) as calculated by GSEA, for represented gene sets from MSigDB for analyses of CAA compared to HGIN and COSCC compared to HGIN. Gene sets differentially enriched or depleted (i.e., FDR q-value < 0.05) in CAA compared to HIGN appear in red. The scatterplot on the bottom right depicts the predicted status of biologically relevant pathways based on z-scores calculated by IPA; groups predicted to be activated (i.e., Z-score > 0) in CAA compared HIGN appear in red. For all graphs, some of the most relevant gene sets or pathways are labeled. Abbreviations: *HGIN* healthy gingiva, *OSCC* canine oral squamous cell carcinoma, *CAA* canine acanthomatous ameloblastoma.

To gain mechanistic insights, Gene Set Enrichment Analysis (GSEA)^29^ and Ingenuity Pathway Analysis (IPA, QIAGEN Inc.) software were applied to all three pairwise comparisons. The MSigDB Hallmark, C2 Canonical Pathways and C6 Oncogenic Signature gene set collections were used for GSEA^30^, while IPA was used to identify biological pathways and molecules predicted to be activated (Fig. 4, Supplemental Tables S7–10). Notable enriched gene sets in CAA and COSCC when compared to HGIN included those associated with oncogenic *KRAS* signaling, epithelial-to-mesenchymal transition (EMT), extracellular matrix (ECM) protein production, angiogenesis, and inflammation. Relevant biological pathways predicted to be activated included RAS, ERK1/2, P38 MAPK, JNK, PI3K, AKT, NFKB, VEGF, TGFB.

### Immunohistochemistry (IHC)

**Figure 5:**
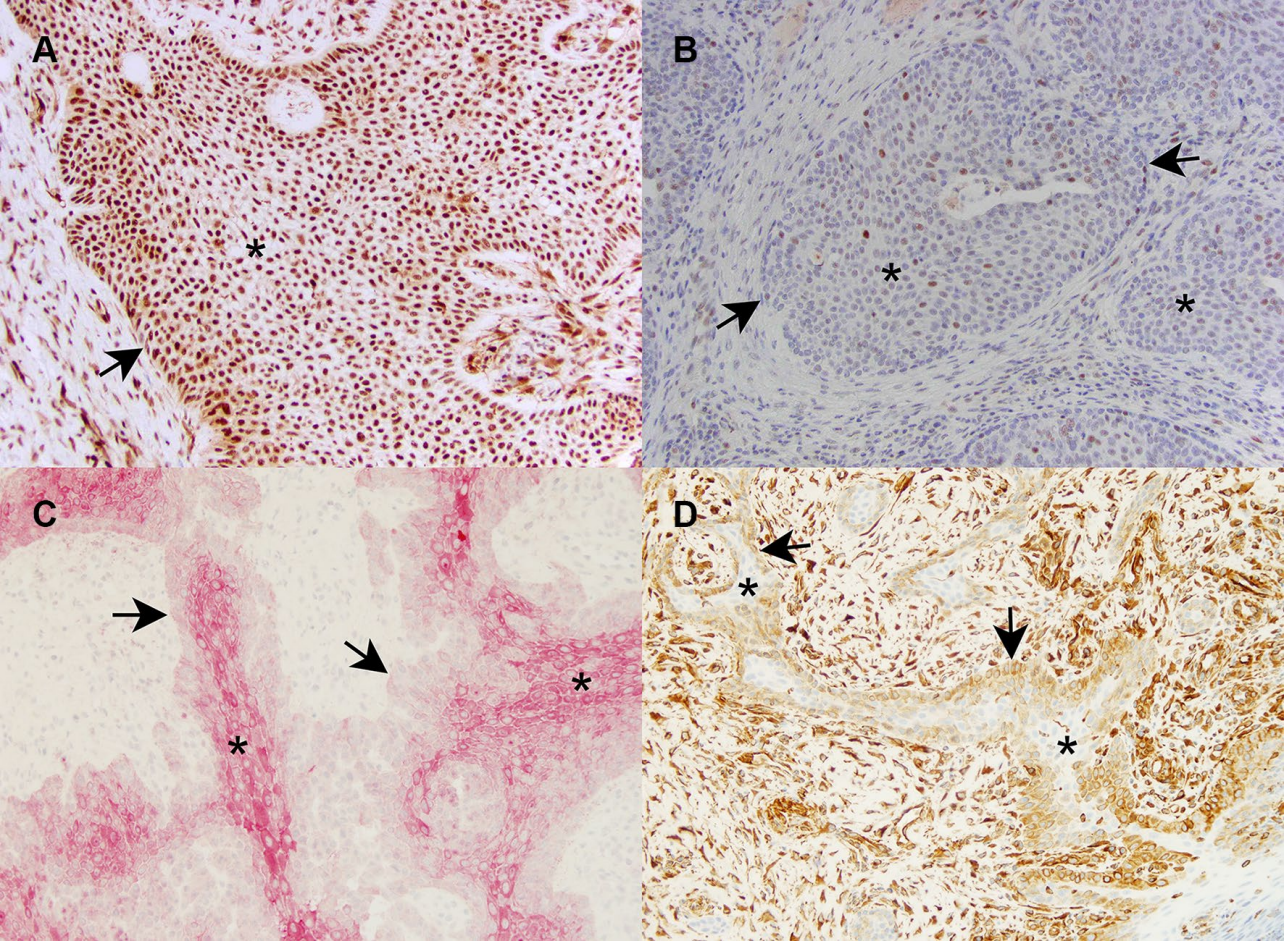
Immunohistochemical validation of MAPK pathway activation and EMT. The asterisks indicate neoplastic cells and the arrows indicate tumor margins. Panels (**A**) and (**B**) are representative photomicrographs of pERK1/2 in CAA (panel **A**, case 15) and COSCC (panel **B**, case 22). Strong and diffuse intranuclear immunolabeling in the neoplastic cells are present in CAA with similar labeling present in stromal cells (**A**). In COSCC, multifocal intranuclear labeling is present in the neoplastic cells at more variable intensity levels (**B**). Panels (**C**) and (**D**) are representative photomicrographs of CK (panel **C**, case 4) and VIM (panel **D**, case 2) immunoreactivity of CAA (IHC, 20X original magnification). Note a gradual increase in cytoplasmic CK expression of epithelial cells from the periphery to the center of neoplastic islands, while the mesenchymal stroma shows no immunoreactivity. Reduced cytoplasmic CK expression along the basal epithelial cell layer at the periphery of neoplastic islands corresponds to cytoplasmic VIM expression, similar to the mesenchymal stroma.

In order to validate the functional enrichment analyses, we performed phosphorylated ERK1/2 (pERK1/2) IHC, as well as pan-cytokeratin (CK) and vimentin (VIM) IHC, to demonstrate downstream MAPK pathway activation and EMT reprogramming of tumor cells, respectively. In the test samples, immunoreactivity for pERK1/2 was multifocal to diffuse with variable nuclear and cytoplasmic expression in neoplastic cells and to a lesser degree, in stromal cells, in some cases (Fig. 5A, B). Loss of CK expression (Fig. 5C) and gain of VIM expression (Fig. 5D) in tumor cells localized along the basal cell layers at the periphery of the islands and cords of neoplastic epithelium, consistent with EMT at the invasive fronts of the neoplasm.

### Molecular homology with AM and dental identity of CAA

**Figure 6:**
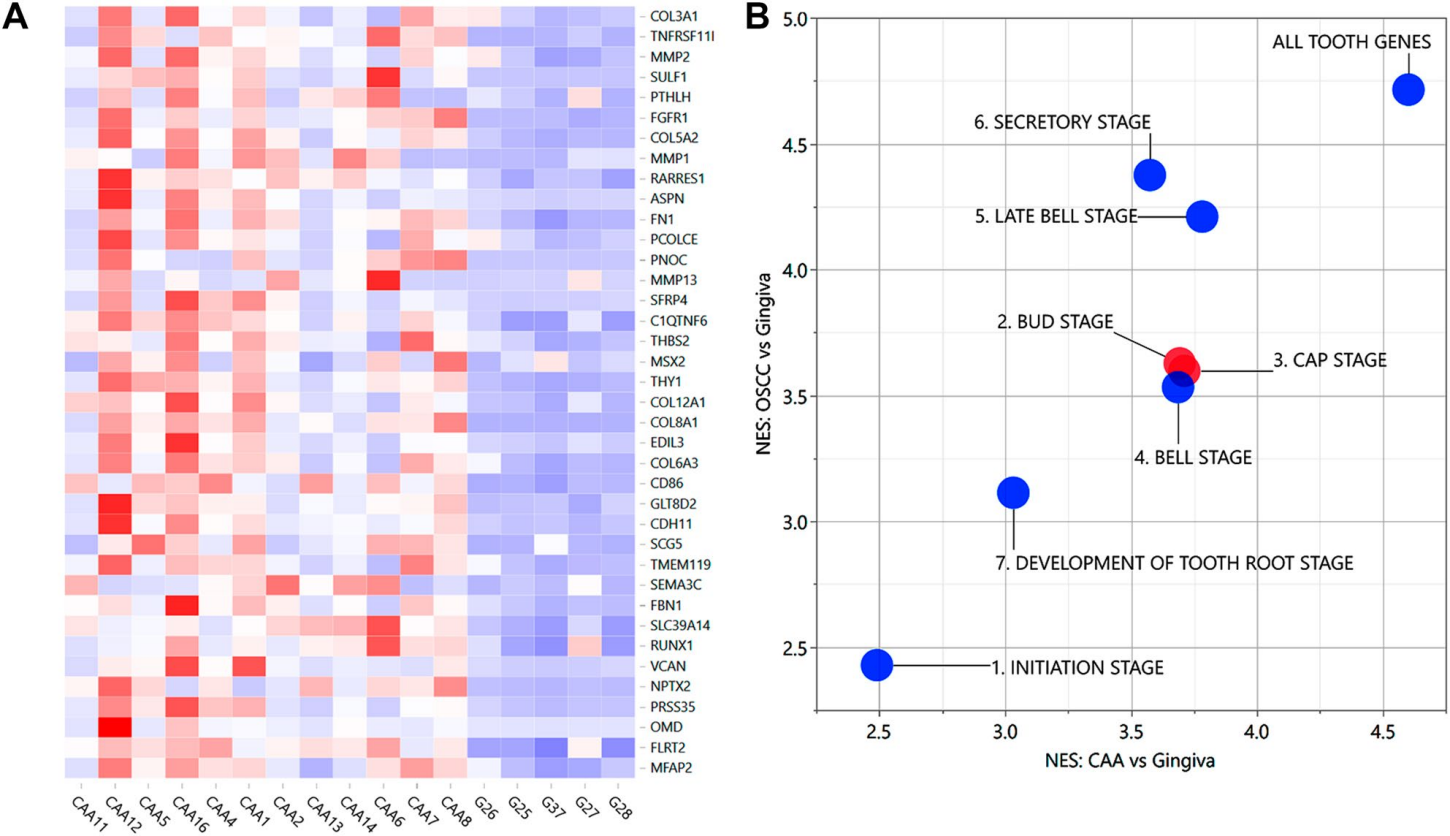
Homology of CAA with AM and dental identity. Panel (**A**) shows a heat map depicting the relative expression of the top 50% leading-edge genes revealed by custom GSEA testing for enrichment of a set of genes known to be upregulated in AM^31^. The heat map shows clear differences between CAA and control samples, and to a much lesser extent, variation among CAA samples. Panel (**B**) shows a scatterplot depicting normalized enrichment scores of custom gene sets of genes known to be involved during different stages of tooth development analyzed with GSEA for CAA relative to HGIN and COSCC relative to HGIN. Red points correspond to gene sets that were significantly more enriched in a separate GSEA analysis of CAA compared to COSCC, and blue points to gene sets significantly more enriched in COSCC compared to CAA. Abbreviations: *CAA* canine acanthomatous ameloblastoma, *COSCC* canine oral squamous cell carcinoma.

To investigate the extent of molecular homology between CAA and AM, GSEA was performed with custom gene sets derived from previously published sets of genes altered in AM when compared to healthy human gingiva^31^. CAA had significant enrichment of corresponding genes known to be upregulated in AM, with most qualifying as leading-edge including *PTHLH*, *FGFR1*, *MMP1*, *MMP2*, *MMP13*, and *COL8A1* (Fig. 6A, Supplemental Table S11). Similarly, a negative enrichment score was observed for genes known to be downregulated in AM.

To determine whether CAA tumors overexpress genes known to be active during specific stages of tooth development, GSEA was performed with custom gene sets associated with the initiation, bud, cap, bell, late bell, secretory, and root development stages, as well as a pool of all 303 known dental genes (http://bite-it.helsinki.fi; Fig. 6B, Supplemental Table S12)^32^. When compared to HGIN and COSCC, CAA exhibited a strong dental identity with significant enrichment of genes associated with the cap and bud stages of tooth development. Some of the related genes noted to be overexpressed in CAA compared to HGIN were *PITX2*, *MSX2*, and *RUNX1*.

## Discussion

To further understand the molecular pathogenesis of CAA and characterize the extent of homology with AM, we explored the expression profile of CAA using a bulk RNA-seq approach and validated selected proteins by IHC. We found that the expression profile of CAA is dominated by signatures of aberrant RAS signaling and involves activation of the EMT cellular program. Moreover, CAA exhibits a strong dental identity and is enriched with genes known to be upregulated in AM^31–33^. The data suggest a high level of molecular oncogenic program homology of CAA and AM.

The prominent role of RAS signaling in CAA was characterized by enrichment of genes known to be upregulated by oncogenic *KRAS* alleles, and by the predicted activation of canonical and non-canonical MAPK pathways including ERK1/2, p38 MAPK, and JNK^34^. The predicted activation of the PI3K-AKT pathway also coincides with this trend given that aberrant RAS signaling frequently underlies dysregulation of this pathway^35,36^. The finding that RAS signaling contributes to CAA is consistent with its mutational landscape, which includes highly recurrent *HRAS* p.Q61R somatic mutations^5,16^. Given that *HRAS* p.Q61R is one of several mutations that can constitutively activate RAS^36,37^, the results of the present and previous studies^13,16^ suggest that this hotspot mutation acts as a driver in CAA. Interestingly, although not found among the most highly enriched pathways in this study, canonical WNT signaling has been previously documented in CAA using IHC^38^. Such findings would be expected in the context of our results given the close interactions that occur between the WNT and RAS-RAF-MAPK pathways^39^.

Activation of the EMT program in CAA correlates with overexpression of *ZEB1* and *ZEB2*, which are well known transcription factors that regulate this cellular process^40^. Similarly, the pattern of upregulation of mesenchymal markers (e.g., *VIM*)*,* extracellular matrix proteins (e.g., *COL8A1, FN1*), and metalloproteinases (e.g., *MMP1, MMP2, MMP13*), and downregulation of cytokeratins (e.g., *KRT3, KRT10*) are typical features of EMT^41^. It is well established that EMT is a dynamic and reversible process that confers neoplastic cells the phenotypic plasticity required for invasion and migration^40,41^. Therefore, these findings were not surprising considering the locally invasive nature of CAA. Additionally, results align with previous studies of AM^42–44^, further suggesting similarities in molecular oncogenesis with CAA. Likewise, given that ERK1/2, TGF beta and TNF are among the pathways that can activate EMT^41,45^, these results underscore the impact and complexity of the cellular reprograming events that take place when aberrant or dysregulated signaling ensues.

The remarkable similar behavior of CAA genes that are known to be up- and down-regulated in AM^31^ demonstrate that canine and human ameloblastomas exhibit a high degree of transcriptional homology. Unsurprisingly, the functional enrichment patterns observed in CAA were comparable to those reported in AM, including notable enrichment of some of the same oncogenic gene sets^31^. Similarly, the strong dental identity observed in CAA is consistent with the reported expression profile of AM^32^, including upregulation of *PITX2* and other transcription factors known to regulate the early stages of tooth development^46,47^. Together with the known clinical, radiographic, histological, and mutational similarities^5,16,22^, these findings support leveraging companion dogs with natural disease as a pre-clinical model of AM. Dogs with naturally-occurring CAA would allow rapid investigations into alternative standalone or combined therapeutic modalities that could complement or substitute traditional surgical approaches. Indeed, aside from RAS-RAF-MAPK signaling, several of the other pathways and molecules identified may represent potential therapeutic targets, including PI3K-AKT, metalloproteinases, and VEGF^48–50^. Additionally, the current dataset could be used to investigate other therapeutic strategies, including those related to tumor metabolism. For example, the glutaminase regulators JUN and NFKB^51,52^ were both predicted to be active; given that glutaminase is a key metabolic enzyme in many types of tumors^51–53^, and has been tied to EMT in several contexts^54–57^, this could justify exploring glutaminase inhibitors as a potential targeted approach for the treatment of CAA.

In this study, we elected to also compare the expression profiles of CAA and COSCC given that they represent the two most common oral tumors of epithelial cell origin in dogs, and because RAS-RAF-MAPK activating mutations occur at detectable frequencies in both tumor types making any transcriptional comparisons potentially insightful^3–5,16,58^. As would be expected based on fundamental differences in biologic behavior^5^, sample clustering analyses showed that CAA and COSCC represent molecularly distinct oncogenic processes. Of note, the expression trends observed in COSCC appeared comparable to those previously reported^59,60^, further confirming the validity and reproducibility of the RNA-seq dataset. Functional analyses showed widespread similarity between CAA and COSCC compared to HGIN, with COSCC typically showing stronger pathway activation. The differences may point to the important clinical and morphological distinctions between the tumor types including benign versus malignant behavior.

Some representative differences between the gene expression profiles of the two tumor types included relative enrichment of genes associated with hypoxia in COSCC. This finding might be explained by the lower proliferation activity, and thus, slower tumor growth found in CAA when compared to COSCC^5^, which likely account for the relatively noticeable infrequent ischemic necrosis in CAA. Another notable functional difference was a more profound predicted loss of PTEN signaling in COSCC compared to CAA. Since PTEN signaling regulates the PI3K pathway^61^, this finding coincides with the higher predicted activation score of PI3K-AKT activity in COSCC. Also, considering the high frequency of *PTEN* and *PIK3CA* mutations in human OSCC^62,63^, such findings justify investigating the mutational status of these pathways in COSCC and CAA.

Apart from functional insights, our RNA-seq data also revealed interesting patterns of expression of a few individual genes. For example, coinciding with human tumors, *SPP1* was significantly overexpressed in CAA when compared to HGIN. Osteopontin (OPN), encoded by *SPP1,* is a multifunctional sialoprotein involved with many important biological processes including cell survival via integrin signaling and PI3K-AKT activation, and EMT regulation^64–66^. Not surprisingly, *SPP1* was differentially overexpressed in COSCC compared to CAA, underscoring known differences in biologic behavior^5^. In line with several studies that have investigated the expression levels of *SPP1* and OPN in different types of cancer in people^65,67,68^, these findings might support the use of *SPP1* or OPN expression as prognostic biomarkers in CAA and COSCC.

Another interesting differentially expressed gene was *ODAM*, which encodes a secreted protein normally produced by mature ameloblasts during the secretory stage of tooth development, as well as by junctional epithelium associated with erupted teeth^26–28^. In this context, the overexpression of *ODAM* in all but one of the CAA tumors is consistent with its dental origin. However, *ODAM* has also been shown to be expressed in several types of cancer in people including gastric, lung and breast tumors^28^, so its expression in CAA could also be secondary to other cellular reprogramming events unrelated to the enrichment of genes associated with tooth development. Regardless, the differential expression pattern of *ODAM* in CAA compared to COSCC might justify investigating it as a biomarker.

It should be noted that, despite clustering together, variation was observed in the expression profiles of samples within each group. This was expected given that samples were obtained from different anatomical sites from individuals of different demographic characteristics, and during potentially different stages of disease. Additionally, given that bulk RNA-seq lacks single cell resolution^69^, and that tissue and tumor samples are an heterogeneous collection of cell types whose exact relative content cannot be controlled, variation in the expression profile is inevitable. This is further compounded by the molecular complexity and dynamism, and genetic diversity of neoplasms in general.

We conclude that the molecular pathogenesis of CAA is largely dominated by aberrant RAS signaling, and involves activation of pathways and cellular programs known to play a role in tumorigenesis including canonical and non-canonical MAPK, PI3K-AKT, EMT, TGFB, and VEGF signaling, and altered ECM production; and that it exhibits a very high degree of homology with AM at the transcriptional level including a strong dental identity.

## Methods

### Clinical samples

The FFPE tissues used in this study were archived by the Anatomic Pathology Section at Cornell University’s College of Veterinary Medicine. Histological assessment of tissues was done using routine H&E-stained samples by a board-certified veterinary pathologist (GED); tumors were diagnosed following previously described criteria while blinded to molecular assays^70,71^. Cryopreserved samples were collected during standard-of-care surgical procedures and stored by the Cornell Veterinary Biobank until retrieved for analysis. Sample collection and experimental procedures were performed in accordance with a protocol (#2005-0151) approved by Cornell University’s Institutional Animal Care and Use Committee. Accordingly, informed consent to authorize the use of tissue samples and clinical data for research purpose was obtained from dog owners prior to sample collection, and undue harm was never inflicted to client-owned dogs for the purposes of this study; all methods were performed in accordance with the relevant guidelines and regulations.

### RNA isolation, library preparation and sequencing

RNA was isolated and poly-A RNA-seq libraries were generated as previously described^16^. Briefly, cryopreserved tissues were homogenized in Trizol (Thermo Fisher) using a bead mill without thawing. RNA was isolated following the manufacturer’s protocol with the following modifications: an extra chloroform extraction was added prior to precipitation, 1 μL glycoblue (Thermo Fisher) was added immediately prior to precipitation, and the RNA pellet was washed twice with 75% ethanol. RNA concentration was measured with a Nanodrop (Thermo Fisher) and integrity determined with a Fragment Analyzer (Advanced Analytical). PolyA + -enriched RNA-seq libraries were generated with the NEBNext Ultra II Directional library prep kit (New England Biolabs) using 250 ng input total RNA. Single-end 85 nt reads were generated on a NextSeq500 instrument (Illumina).

### RNA-seq analysis

Raw reads were trimmed for low quality and adaptor sequences and filtered for minimum length with cutadapt software (parameters: -m 20 –q 20 -a AGATCGGAAGAGCACACGTCTGAACTCCAGTC—match-read-wildcards)^72^. Trimmed reads were mapped to the reference genome/transcriptome (Ensembl CanFam3) using tophat v2.0 (parameters—no-novel-juncs—library-type fr-firststrand—G < Ensembl_CanFam3_genes.gtf >)^73^. Differential gene expression was analyzed with cufflinks v2.2^74^; additional filters included defining ‘expressed’ genes (minimum avg(FPKM) > 10 in at least one group) and ‘stringent differentially expressed genes’ (‘expressed’ genes with FDR < 0.05 and minimum twofold change between groups). Clustering analyses were generated in R v3.6.1 using variance-stabilized counts from DEseq2 v1.26.0 and the functions prcomp (principal components analysis) and hclust (hierarchical clustering with Euclidean distance and ward.D criteria). The canine gene symbols were converted to human gene symbols using Biomart (Ensembl) one-to-one orthology assignments for protein-coding genes to enable analysis of MSigDB gene sets with GSEA. GSEA pre-ranked analysis included the Hallmark, C2: Canonical Pathways, and C6: Oncogenic Signature gene sets using log2-fold change values for ‘expressed’ genes for each pairwise comparison with the classic enrichment statistic^75^. The heatmap of leading-edge genes was generated in R from row-normalized FPKM values with d3heatmap v0.6.25.Cuffdiff output files were converted to human gene identifiers and filtered for one-to-one orthologs (Ensembl Biomart) and min FPKM = 1, and uploaded to Ingenuity Pathway Analysis (Qiagen) for pathway analysis.

### qPCR validation

The levels of expression of a subset of differentially expressed genes were validated using real-time reverse transcription polymerase chain reaction (qPCR). cDNA was synthesized as previously described^16^. All cDNA reactions were diluted 20-fold with water prior to qPCR reaction setup. Primer pairs were designed with Primer-BLAST (NCBI), separated by an intron to minimize amplification of residual contaminating genomic DNA and allow identification of alternate amplicons with melt curve analysis. *RPL13A* was selected as the endogenous control gene, as this gene showed minimal variation across samples in the RNA-seq data^76^. Each primer pair was validated using a standard curve of six four-fold serial dilutions of a representative sample of pooled cDNA. A ‘No-RT’ control containing RNA but lacking M-MuLV enzyme and one ‘no template’ control lacking any cDNA sample was included for each primer pair standard curve validation. Primer pairs that did not generate signal in < 35 cycles or that exhibited non-quantitative performance (i.e., < > 2-cycle shifts for fourfold dilution series), non-specific signal in negative controls, or variable amplicon identities as determined by melt curve analysis were excluded. All of the primer pairs in Table 2 passed validation by standard curve testing. Each qPCR reaction was prepared in 8 µL reaction volumes in an optically clear 384-well PCR plate with seal using the Luna Universal qPCR Master Mix (New England Biolabs) with 0.25 µM primers and 4 µL pre-diluted sample cDNA. All reactions were performed in triplicate using a Roche LightCycler 480 instrument. Cycles were as follows: initial incubation 5 min at 95 °C; followed by 45 cycles of 30 s at 95 °C; 30 s at 60 °C; 10 s at 72 °C with data acquisition; and final a melt curve with a ramp from 60 to 95 °C at 2 °C per second. Melt curve analysis was used to identify and exclude reactions with alternative amplicons. For relative quantification estimates for each target gene, the ΔΔCt value [ΔCt_SAMPLE_—ΔCt_REF_] was calculated for each sample, where ΔCt_SAMPLE_ = average (target gene Ct)—average (all endogenous control Ct) and ΔCt_REF_ was defined as the average ΔCt_SAMPLE_ for the normal samples. The normalized relative amount of the target gene is 2 − ΔΔCt^77^.

**Table 2:** Primer pairs used for qPCR.

Target gene	Forward (5′ → 3′)	Reverse (5′ → 3′)	Product (bp)
COL8A1	AAGCGGCACCTAAGAAAGGC	TTTGGTTCCAGGTATCCCATGAC	134
IL13RA2	TGGGTCATCAGAATCCCAGC	AGGTAGTCTGGTGGCATAGG	87
KRT3	CAAGTGAAGACCCAGGAGCG	TGTGATGGAATTTGTGCCTTGC	150
MSX2	CAGGAGCCCGGCAGATACTC	TCTCGGCTTCCGATTGGTC	90
ODAM	AAGGCCAAGACTGATTACTTAAAGG	TGTTAGCATCGAGGAATCAAATG	124
PNOC	ATATGCTGGTGTGGCTGGTACG	GAGCAGCAGGAGGTCACAAAG	136
SFRP2	AGGATGACAACGACATAATGGAAAC	GTCTTGCTCTTGGTCTCCAGG	121
SPP1	TGACCCATCTCAGAAGCAGAC	TCGTCATGGCTTTCATTGGAC	114
SULF1	TGCAACCCAAGACCTAAGGG	ACCTTCCCATCCATCCCATAAC	93
TNFRSF11B	GCGACACAGCTCACAAGAAC	ACGCTGTTCTCACACAGGTC	117
WIF1	GCCAATGTCAAGAAGGCTGG	TTAAGTGAAGGCGTGTGTTGC	115
RPL13A*^76^	GCCGGAAGGTTGTAGTCGT	GGAGGAAGGCCAGGTAATTC	87

Genes, primer pairs, and product size (bp = base pairs) used for qPCR analysis.

*Endogenous control gene.

### Immunohistochemistry (IHC)

Selected FFPE tissue blocks from each case were processed for antigen retrieval and detection by using an automated IHC processor (Leica Bond-Max, Leica Biosystems, Buffalo Grove, Illinois, USA), as previously described^5^. Briefly, sections were dewaxed (cat# AR9222, Bond Dewax Solution, Leica) and processed for epitope retrieval (cat# AR9961 or AR9640, Bond Epitope Retrieval solution, Leica) followed by incubation with the primary antibody. For VIM, the mouse monoclonal IgG2a, kappa anti-vimentin, clone Vim 3B4 (cat# M7020, Agilent Dako, Santa Clara, California, USA) was used. For pERK1/2, a rabbit monoclonal (EPR19401) to ERK1 (phospho T202) and ERK2 (phospho T185) (cat# ab201015, Abcam, Cambridge, United Kingdom) was used. For CK, a mouse monoclonal anti-human clone AE1/AE3 antibody (cat# M3515, DakoCytomation, Carpinteria, California, USA) was used. Next, polymeric alkaline phosphatase conjugated anti-mouse IgG (cat# PV6110, PowervisionTM Poly-AP Anti-Mouse IgG, Leica) was applied followed by Red DetectionTM (cat# DS9390, Bond Refine Red Detection Kit, Leica), and hematoxylin counterstain. For ODAM, FFPE tissue sections were manually deparaffinized in xylene, rehydrated in graded ethanol, and subjected to antigen retrieval by steaming in citrate buffer (10 mM, pH6.0) for 20 min. Then, endogenous peroxidase activity was quenched with 0.3% hydrogen peroxide followed by incubation with rabbit polyclonal anti-ODAM IgG (cat# orb317695, Biorbyt Ltd., Cambridge, UK) diluted 1:300 overnight at 4 °C and 5 h at room temperature followed by incubation with ImmPRESS HRP Anti-Rabbit Ig (Peroxidase) Polymer Detection Kit (Vector Laboratories), Nova Red chromogen (Vector Laboratories), and hematoxylin counterstain. In each assay, a positive control reference tissue was included on the same slide as the sample. Negative controls consisted of a duplicate tissue section of each case incubated with an isotype-matched irrelevant primary antibody. Scoring of pERK1/2 was performed by a board-certified veterinary pathologist (ADM) by defining the following variables: distribution (absent, multifocal, or diffuse), pattern (cytoplasmic or nuclear), labeled cells (neoplastic or stromal), and intensity (none, weak, intermediate, or strong). Scoring of VIM and CK were performed by a board-certified veterinary pathologist (GED) using the same criteria.

## Supplementary Information

Supplementary Tables.

## Data Availability

The gene expression data is available at the NCBI Gene Expression Omnibus (GEO) with accession number GSE175876. All other relevant data is included in the paper.
